# Early Experience of Helical Tomotherapy for Hepatobiliary Radiotherapy

**DOI:** 10.1155/2011/545267

**Published:** 2011-09-15

**Authors:** Carole Massabeau, Virginie Marchand, Sofia Zefkili, Vincent Servois, François Campana, Philippe Giraud

**Affiliations:** ^1^Department of Radiation Oncology and Medical Physics, Institut Curie, 75005 Paris, France; ^2^Department of Radiation Oncology, Institut Claudius Regaud, 31052 Toulouse, France; ^3^Department of Radiology, Institut Curie, 75005 Paris, France; ^4^Department of Radiation Oncology, European Georges Pompidou Hospital, 75015 Paris, Paris Descartes University, 75005 Paris, France

## Abstract

Helical tomotherapy (HT), an image-guided, intensity-modulated, radiation therapy technique, allows for precise targeting while sparing normal tissues. We retrospectively assessed the feasibility and tolerance of the hepatobiliary HT in 9 patients. A total dose of 54 to 60 Gy was prescribed (1.8 or 2 Gy per fraction) with concurrent capecitabine for 7 patients. There were 1 hepatocarcinoma, 3 cholangiocarcinoma, 4 liver metastatic patients, and 1 pancreatic adenocarcinoma. All but one patient received previous therapies (chemotherapy, liver radiofrequency, and/or surgery). The median doses delivered to the normal liver and to the right kidney were 15.7 Gy and 4.4 Gy, respectively, below the recommended limits for all patients. Most of the treatment-related adverse events were transient and mild in severity. With a median followup of 12 months, no significant late toxicity was noted. Our results suggested that HT could be safely incorporated into the multidisciplinary treatment of hepatobiliary or pancreatic malignant disease.

## 1. Introduction

Majority of patients who develop either liver malignancy (metastasis or hepatobiliary primaries) have unresectable disease [1, 2]. Moreover, after first resection of liver lesions, recurrences are observed in two thirds of patients despite the use of systemic chemotherapy. In recent years, several new methods of nonsurgical ablation of liver malignancies have been tested, such as radiofrequency ablation, but also cryotherapy, laser hyperthermia, intra-arterial therapies, or ethanol injection, with variable success [3]. Until recently, radiotherapy of hepatic malignancies was playing a limited role due to the well-known limited radiotolerance of the liver [4, 5]. Recently, there is an increasing interest for modern radiotherapy as an attractive alternative, because it is noninvasive and not limited by anatomical issues associated with other therapies as the size, multiplicity and location of liver lesions [6]. New radiation techniques including intensity modulated radiation therapy (IMRT), image-guided radiation therapy (IGRT), and stereotactic radiosurgery make it possible to deliver optimally high doses to the target volume with minimal effect on adjacent radiosensitive tissues. 

Literature concerned with modelling liver tolerance indicate that high doses of radiation therapy can be delivered without significant toxicity, as long as a certain amount of normal liver is spared [7, 8]. The liver parenchyma is composed of numerous functional subunits that tolerate substantial focal injury prior to any clinical sequelae. Partial liver irradiation to high doses is, consequently, possible if adequate normal liver parenchyma can be spared [9]. The risk of RT-induced liver disease (RILD) is increasing with the preexisting liver disease and with dosimetric parameters among which the most important were identified to be the mean liver dose and the volume of liver receiving more than 30 Gy [10]. With the development of new technologies and techniques, we are able to focus the radiation more precisely on the lesion to provide a higher dose to the tumor [11, 12]. Helical tomotherapy (HT), an image-guided, intensity-modulated radiotherapy system, can allow for simultaneous and precise targeting of multiple lesions, while sparing normal tissues [13]. The objective of the current study was to review our initial experience using HT for irradiation of hepatobiliary malignant disease.

## 2. Cases Presentation

### 2.1. Patients and Treatment

Between May 2008 and July 2010, 9 patients who underwent a course of HT (Hi-Art system, TomoTherapy, Madison, wis, USA) in the Radiation Department of the Institut Curie for malignant hepatic lesions entered in our study. The baseline characteristics of the nine enrolled patients as well as the treatment details are listed in Table 1. A total of 7 patients received chemotherapy prior to irradiation, 5 underwent previous hepatic surgery, and 2 underwent previous radiofrequency ablation. Patient 7 was a 63-year-old man whose hepatitis B-related hepatocarcinoma (Child-Pugh class A disease) was initially treated with a left lobectomy. When a multifocal recurrence occurred, both chemotherapy and two segmental liver resections were performed, leading to one year of clinical remission before another local recurrence, presented as a single 3.6 cm lesion. Since the location of this lesion (directly adjacent to the median hepatic vein) precluded surgical management and RF ablation, patient 7 was referred for the HT. Two of the 3 cholangiocarcinoma patients were treated in a curative intent in a neoadjuvant setting according to the Mayo Clinic protocol. This is a protocol combining neoadjuvant concurrent chemoradiotherapy with capecitabine and cadaver donor liver transplantation for patients with operatively confirmed stage I and II hilar cholangiocarcinoma. Concurrent capecitabine which could be used as an irradiation sensitizer, was started on the first day of irradiation, half in the morning and half in the evening, for the duration of the radiation therapy [14]. All patients provided written informed consent before HT started. HT was performed to deliver the prescribed dose in 27 or 30 daily fractions, 5 days a week. Before each treatment, a megavoltage CT scan in the HT Hi-Art system was made to adjust table position and to verify the position of the tumor and vital organs.

### 2.2. Tomotherapy Planning

Patients were immobilized for initial simulation and for treatment with the two arms above the head in a body frame. Simulation was performed in a large bore computed tomography (CT) (Aquilion LB, Toshiba medical, Puteau, France SA) of 90 cm aperture. Images were acquired with 3-mm slice thickness from the mid-neck to the pelvis. Intravenous contrast was used to facilitate the appreciation of the tumour volume. Planning images are obtained by a four-dimensional CT (4D-CT) to assess respiratory motion. Regular breathing can lead to organ motions up to several centimeters which are taken into account by adding a specific margin around the target volume. The CT data was transferred to a linac-based planning system (Eclipse 3D version 8.1; Varian Medical Systems Inc., Palo Alto, USA) for delineating target volume and organs at risk (OAR). The gross tumour volume (GTV) was contoured manually corresponding to the tumour volume seen in the CT scan and in the co registered MRI images [17]. No specific size limit was placed on the tumor diameter. A margin of 1 cm to account for microscopic disease extension was added to the GTV to define the clinical target volume (CTV). An additional safety margin for liver movement caused by breathing and other nonspecific setup error was placed around the CTV to define the final planning target volume PTV (PTV) [18, 19]. For the OAR, the entire liver, the kidneys, the spinal cord, and the lungs were outlined. The normal liver was defined as the total liver minus the GTV. The CT images and accompanying contours were exported to the HT planning system (HiART Version 2, Tomotherapy Inc., Madison USA) for planning. According to the International Commission on Radiation Units and Measurements reports [20], the dosimetric planning objectives consisted of achieving full uniform dose coverage of the target, while keeping the dose to critical structures below their tolerance. The PTV must receive between 95% and 107% of the prescribed dose. For organs at risk (OAR), the dosimetric constraints have been set based on previously published toxicity data reviewed in the QUANTEC recommendations [21]. For partial liver irradiation, the median normal liver dose must be under 28 Gy (in 2-Gy fractions) for primary liver cancer and under 32 Gy (in 2-Gy fractions) for liver metastases. The French guidelines recommended to give less than 26 Gy in the total liver and to restrict to 50% the volume of normal liver receiving 30 Gy or more [16]. The total kidney must receive less than 20 Gy and the mean kidney dose must stay below 18 Gy; the maximum dose to the spinal cord was 45 Gy, and the percentage of the right lung receiving 20 Gy or more, must be limited to 20% (Table 2).

### 2.3. Dosimetric Results

**Figure 1:**
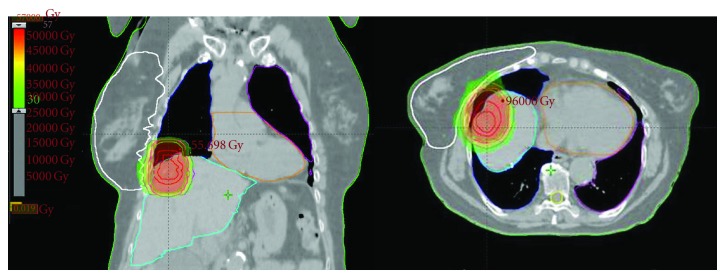
Distribution of isodoses with HT treatment planning in the patient 8 (hepatic dome metastasis) in axial and frontal representation. The different doses as well as the target volumes/organs at risk are represented with different colors. Red color represents the target volume dose (>54 Gy). Green color represents lower radiation doses of 30 Gy.

**Figure 2:**
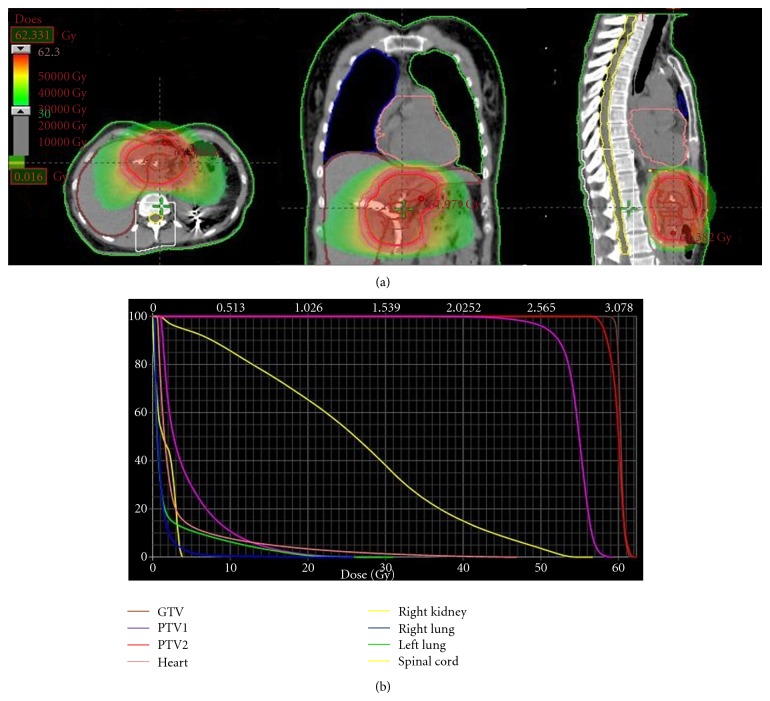
Distribution of isodoses with HT treatment planning in the patient 9 (cholangiocarcinoma) and the corresponding dose-volume histogram. The different doses as well as the target volumes/organs at risk are represented with different colors. Red color represents the target volume dose (>54 Gy). Green color represents lower radiation doses of 30 Gy.

The dosimetric results are listed in Table 3. The doses to the target volumes always met their prescription constraint. The median liver V30 was 12% (6–37.2), well below the 50% recommended limit, while the median liver dose was 15.7 Gy (9.7–25.9) (recommended limit: 28 Gy). The median dose delivered to the right kidney was 4.4 Gy (1.5–9.7) and remained less than the recommended constraint of 18 Gy. The same observation can be made for the lungs, the left kidney, and the spinal cord) (data not shown). The distributions of isodoses for the patient 8 and 9 are shown in Figures 1 and 2.

### 2.4. Acute and Late Toxicities

Toxicities were assessed using the Radiation Therapy Oncology Group/National Cancer Institute Common Toxicity Criteria, version 3 morbidity scale [15], every week during the HT course to address side effects and monitor laboratory values. All but one patient completed the prescribed treatment. One of the two cholangiocarcinoma patient prematurely stopped HT for recurrent cholangitis on day 3rd of the radiation treatment, effectively treated with antibiotics, stent revision and surgery. As shown in Table 4, only minor toxicities developed during treatment. Most of the treatment-related adverse events were transient and mild in severity, with no case of direct treatment-related death. The hematologic and hepatic disorders occurred 1-2 weeks after the start of treatment and regressed spontaneously without interfering with the scheduled delivery of HT. We reported 1 thrombopenia grade 2 leading to 5 days of capecitabine interruption. The thrombopenia was most likely to be related to the capecitabine than the HT.

One patient experienced cytolysis grade 2, ten weeks after the HT course. A persistent thrombopenia grade 2 and cholestasis grade 1 (more than 4 months) occurred in one patient with progressive disease confirmed 4 months after the end of the radiation treatment. No radiation-induced liver disease was reported during the months following the HT.

### 2.5. Disease Outcome

After completing chemoradiotherapy, follow up was performed at 1–3 month intervals thereafter. The tumor response was assessed with follow-up CT scans. At study analysis, all but one patient, who died from progressive disease, were still alive. The median duration of followup after the HT course was 12 months (4–32). The cholangiocarcinoma patient treated in a neoadjuvant setting underwent successful cadaveric liver transplant 3 months after chemoradiotherapy with a complete histological response and remains disease-free 2 years later as the cholangiocarcinoma patient treated in an adjuvant setting. The pancreatic adenocarcinoma patient remains disease-free during 10 months then bone metastasis and local progression occurred. The hepatocellular carcinoma patient attained complete clinical remission after the helical Tomotherapy. Two years later, however, this patient experienced lung metastasis and local hepatic progression. Only one in-field progression (progression of disease inside the targeted tumor volume) occurred in the melanoma metastatic patient (patient 1), who died from his progressive disease, while the 2 colorectal metastatic patients developed exclusively out-of-field (patient 8) and distant progression without local relapse (patient 9).

## 3. Discussion

### 3.1. General and Technical Issue

Radiation oncology has seen the development of new technology which offers significant improvements in local control and reductions in toxicity. Increased doses >50 Gy with non-3D techniques improved tumor control marginally but were associated with a relatively high incidence of liver and gastrointestinal toxicity [10, 22]. Recently, the modulated intensity radiotherapy has allowed local radiation to the liver to be performed safely and have yielded promising results for dose escalation [23]. We report here our early experience of the use of the modulated intensity with HT for irradiation of hepatobiliary malignant disease in 9 patients with several clinical settings. The HT was not limited by the tumour size, the tumour location, the multiplicity of lesions as well as a previous history of abdomen irradiation or a preexistant liver disease. Helical tomotherapy, a new type of RT, combines megavoltage computed tomography (CT) imaging with an intensity-modulated RT system. Such a combination allows for the delivery of precise RT to the tumor area while sparing normal tissues. In addition, this system can perform simultaneous RT of multiple lesions during the course of rotational fan beam RT delivery. This device is an ideal tool for delivering multifocal and high-dose radiation treatment and allows the irradiation at different dose levels in a single treatment session. With regard to radiation toxicity, in our study, treatment was feasible, safe, and very well tolerated with only mild and transient clinical or biological adverse effects. No patient developed the clinical syndrome of radiation-induced liver disease (RILD). This syndrome is known to be related to the volume of normal liver receiving more than 30 Gy [15]. By incorporating the analysis of the histogram dose volume in the treatment planning process, algorithms could allow us to better adjust the prescribed dose and regimens [24]. Data from our study indicate that the HT planning achieved to give a highly conformal treatment plan sparing as much normal liver as possible and respecting the recommended dose-volume limits, making it possible to deliver a radiation dose of 54 Gy or more, in a standard regimen (2 Gy per fraction).

### 3.2. HT for Liver Malignancies

*Hepatocellular carcinoma (HCC)* is one of the most common malignant diseases worldwide. Only 10% to 15% of patients are candidates for curative surgery because of the size of the tumour, disease multifocality, early vascular invasion, decompensated liver disease, or poor performance status [25]. Some alternative treatments, such as percutaneous ethanol injection, radiofrequency ablation, and transcatheter arterial chemoembolization, tend to be more effective in small tumours (from <2 cm to 4 cm in greatest dimension) but have some contraindications as they are invasive techniques. No standard treatment has been established in locally advanced HCC [26]. External beam irradiation therapy for HCC has been used infrequently because of the limited tolerance of the entire liver [6, 22, 27]. Case series data published by Dawson et al. [28] have shown median survivals of 11 to 15 months with the use of radiation in unresectable hepatobiliary cancer, which compares favorably with other modalities. Three-dimensional conformal and more recently IMRT has come to be recognized as a potentially option for advanced HCC, since it may enable the safe escalation of the dose to the tumor [28–31]. McIntosh et al. [32] reported initial experience with IMRT (50 Gy in 20 fractions) plus capecitabine for patients who had large HCC lesions, with acceptable toxicity and promising local control. Besides, another approach, the stereotactic body radiation therapy (SBRT) consisted of the delivery of a high tumor dose with an extreme precision in only few fractions of very high dose (10 or 20 Gy per fraction). This represents a promising noninvasive treatment for unresectable small HCC previously successfully tested in liver metastasis [33–37].

Until recently, modern radiation therapies were studied for *liver metastatic* patients by the way of symptom palliation [25]. The liver metastases derived most often from colorectal cancer, whose prognosis has really changed in recent years, suggests the need for an effective local treatment. The resectability rate is reported to be only 25%. The recent spread of interstitial therapies and radiofrequency has further increased the possibilities of liver metastasis treatment [3, 38, 39]. The stereotactic body radiation therapy (SBRT) has been shown to be an effective, well-tolerated treatment but the tumor size might be a limiting factor. Baisden et al. [24] proposed a model based on the planning target volume (PTV) and liver volume to predict the maximum tolerable dose (MTD) delivered to a lesion by HT-based SBRT. Exactly how high the dose should be for each treatment, how many fractions in total are optimal, and how much time should pass between treatments are still to be resolved. For metastatic liver patients with an acceptable performance status, without active systemic disease, we argue that a standard regimen (2 Gy per fraction, during 6 weeks) of the well-tolerated HT could be as interesting alternative in particular for the large and/or multiple lesions.

*Intrahepatic cholangiocarcinoma (ICC)* is a rare hepatic malignancy that for the 30% patients with unresectable disease is uniformly fatal [40]. Systemic chemotherapy has been disappointing in regard to its efficacy, with most regimens resulting in a median survival of 6 to 12 months [41]. There has been great interest in other modalities of treatment, particularly intra-arterial therapies, and conformal radiotherapy, such as IMRT [42–44]. Baisden et al. reported the feasibility and acceptable tolerance of photodynamic therapy and concurrent chemoradiotherapy with HT (50 Gy in 25 fractions) in 10 unresectable hilar cholangiocarcinoma [45]. The most promising results have been achieved with combinations of these techniques, with the use of neoadjuvant chemoradiotherapy prior to orthotopic liver transplantation at the Mayo Clinic [46]. This approach has provided improved histological response as well as a better outcome with a 5-year survival rate higher by 20% to 40%. The use of HT in a neoadjuvant strategy before liver transplantation might increase the tolerance of the chemoradiation course avoiding an excessive adverse event which could interfere with the liver transplantation [47].

### 3.3. HT for Pancreatic Malignancies

The use of HT for *pancreatic adenocarcinoma,* whose prognosis and local control remains a challenging issue for oncologists, has been recently introduced [48]. Indeed, Ji et al. published the early results of a feasibility study as well as the early clinical outcome of concurrent administration of capecitabine with HT in 19 patients with advanced pancreatic cancer [49]. Another basis for offering radiotherapy to patients with pancreatic cancer is palliation of symptoms due to local invasion such as biliary and gastrointestinal obstruction. Because of its ability to restrict the dose to normal organs and minimize radiation toxicities, HT may be an ideal palliative option for challenging cases of pancreatic cancer.

### 3.4. Therapeutic Combinations

The use of high-precision external beam radiotherapy can be complementary or an alternative to other treatments. For example, radiation may be offered to patients with large tumors that exceed the size that can be treated by radiofrequency ablation or surgical resection. The shrinkage of these lesions could enable other local treatment. Moreover, a lesion that is treated by chemoembolization may be found to have an alternate vascular supply that cannot be occluded. Radiation can play a complementary role in these cases and be added to this modality. One study demonstrated that in HCC patients who had failed transarterial chemoembolization, local radiation induced an additional tumor response [50]. The collaboration of surgeons, medical oncologists, radiation oncologists, gastroenterologists, radiologists, and pathologists might offer better therapeutic indexes for challenging cases in a multidisciplinary approach.

## 4. Conclusion

We reported, here, our preliminary experience of the use of HT in various hepatobiliary malignant diseases as a way of understanding the perspectives offered by such a modern radiotherapeutic technique. Further investigations like comparative planning studies and longer followups are needed to confirm the dosimetric and clinical benefits offered by the HT over standard techniques or other new technique such dynamic arc therapy.

##  Conflict of Interests

No potential conflict of interests relevant to this paper was reported.

## Figures and Tables

**Table 1:** Patient and disease characteristics.

Patient	Sex	Age	Performance Status (ECOG)	Primary tumour site	Previous therapies	Location/number/size of liver lesions
1	M	51	0	Rectum Ad.	Partial liver resection CT Pelvic RT RF	Hepatic dome 3 lesions (34; 10; 9 mm)
2	M	42	0	Cholangio	Biliary stent CT	Diffuse periductal infiltration Not measurable
3	F	73	2	Colon Ad.	CT Colon surgery	Posterior to the right portal branch 1 lesion (20 mm)
4	F	60	0	Pancreatic Ad.	CT Biliary Stent Partial liver resection	Pancreatic mass 1 lesion (40 mm)
5	F	72	0	Cholangio.	Extensive hepatobiliary surgery	Hilar region (no macroscopic disease)
6	M	64	1	Colon Ad.	Colon surgery CT	Perihilar metastasis 1 lesion (70 mm)
7	M	63	0	HepatoC.	Left hepatectomy Partial liver resections CT	Adjacent to the hepatic vein trunk (1 lesion-36 mm)
8	F	80	0	Colon Ad.	CT Partial liver resection RF	Hepatic dome 1 lesion (10 mm)
9	F	48	1	Cholangio	No ^a^	Hilar infiltration (20 mm)

Abbreviations: HT: helical tomotherapy; M: male; F: female; Ad: adenocarcinoma; Cholangio: cholangiocarcinoma; HepatoC: hepatocarcinoma; CT: chemotherapy; RT: radiation therapy; RF: radiofrequency ablation  ^a^The patient 9 underwent an abdominal irradiation for Hodgkin lymphoma thirty years ago.

**Table 2:** Dosimetric constraints for each organ at risk: recommended dose-volume limits from Quantec [15] and French guidelines [16].

Normal liver	Median dose <28 Gy (in 2-Gy fractions)
	V30 < 50% ^a^

Right kidney	Maximum dose of 20 Gy to the total kidney
	Mean dose < 18 Gy ^a^
Right lung	V20 < 20% ^a^
Spinal cord	Maximum dose of 45 Gy

^a^V*x*: Percentage of the organ at risk receiving more than *x* dose.

**Table 3:** Treatment characteristics and dosimetric results.

Patients	Radiation dose per fraction (/F)	Concurrent capecitabine (mg·m²·day)	Median dose to the PTV ^a^	PTV ^a^ volume (cc)	Normal liver volume ^b^ (cc)	Normal liver V30 ^c^ (%)	Median normal liver dose (Gy)	Median right kidney dose (Gy)
1	54 Gy 2 Gy/F	no	56.6	417.7	1244	8	25.5	1.7
2	54 Gy 2 Gy/F	1500	57.2	381	1726.2	37	25.9	5.3
3	60 Gy 2 Gy/F	1500	61	268	1424,3	12	12.1	1.5
4 ^d^	54 Gy 60 Gy 2 Gy/F	1500	55.5 ^e^ 61.6 ^f^	671.9 143.5	1653.9	7,5	13.2	9.7
5	54 Gy 1.8 Gy/F	1600	54.1	174.6	892.9	17.5	15.7	4.4
6	54 Gy 1.8 Gy/F	no	54	262.6	857	30	22.1	2.1
7	60 Gy 2 Gy/F	no	61.1	121	1160.1	10	14.3	1.6
8	54 Gy 2 Gy/F	1500	54	93.9	1272.3	6	9.7	4.5
9 ^d^	54 Gy 60 Gy 2 Gy/F	1500	54 ^e^ 59.8 ^f^	275.9 221.7	1484.1	37.2	25.6	4.5

^a^PTV: planning target volume;  ^b^Normal liver volume: liver volume minus PTV;  ^c^V30: percentage of the normal liver receiving 30 Gy or more;  ^d^For patient 4 and patient 9, two levels of dose were prescribed: 54 Gy to the PTV 1 (gross tumour volume (GTV) + 2 cm margin) and 60 Gy to the PTV2 (GTV + 1 cm margin);  ^e^Median dose to the PTV1;  ^f^Median dose to the PTV.

**Table 4:** Acute clinical and biological adverse events: maximum toxicity grade per patient (Radiation Therapy Oncology Group/National Cancer Institute Common Toxicity Criteria, version 3) [15].

Patient	Nausea	Pain	Diarrhea	Fatigue	Thrombopenia	Cytolysis	Cholestasis
Patient 1	0	0	0	0	1	1	1
Patient 2	0	0	0	1	2	0	1
Patient 3	0	0	0	1	0	0	1
Patient 4	0	0	0	0	0	0	0
Patient 5	1	0	0	1	0	0	0
Patient 6	0	0	0	0	0	0	0
Patient 7	0	0	0	0	1	0	0
Patient 8	0	0	0	1	1	0	0
Patient 9 ^a^	1 ^a^	1 ^a^	0	1 ^a^	0	1 ^a^	4 ^a^

^a^Symptoms were not related to the HT but most likely to biliary stent obstruction with cholangitis, which is a major concern in cholangiocarcinoma patients.
